# Measuring the constructs of health literacy in the Iranian adult Kurdish population

**DOI:** 10.1186/s12889-021-10589-z

**Published:** 2021-03-24

**Authors:** Arezoo Yari, Marzieh Soofimajidpoor, Ghobad Moradi, Farzam Bidarpoor, Haidar Nadrian, Abedin Iranpoor, Mehdi Zokaie, Daem Raoshani, Nahid Ghotbi, Yadolah Zarezadeh

**Affiliations:** 1 Social Determinants of Health Research Center, Research Institute for Health Development, Kurdistan University of Medical Sciences, Sanandaj, Iran; 2 Social Determinants of Health Research Center, Tabriz University of Medical Sciences, Tabriz, Iran; 3 HIV/STI Surveillance Research Center, WHO Collaborating Center for HIV Surveillance, Institute for Futures Studies in Health, Kerman University of Medical Sciences, Kerman, Iran; 4 School of Health, Kurdistan University of Medical Sciences, Sanandaj, Iran; 5 Department of Epidemiology and Biostatistics, Medical School, Kurdistan University of Medical Sciences, Sanandaj, Iran

**Keywords:** Construct, Health literacy, Kurdish population

## Abstract

**Background:**

Health literacy is essential to self-care, which is an important precedence to improve the quality of healthcare services and a key factor in health. It also plays a pivotal role in decision-making in various health fields. Therefore, policymakers consider health literacy to be a primary tool to promote community health and enhance the proper use of healthcare services. The present study aimed to assess the health literacy status of the Kurdish population in Kurdistan province, Iran based on the nine constructs of the Iranian health literacy questionnaire (IHLQ) individually and collectively and determine the significant effects of demographic variables on health literacy.

**Methods:**

This cross-sectional study was conducted on the Iranian adult Kurdish population living in the urban and rural areas of Kurdistan province, willing to participate during April 2017–September 2018. Data were collected using the IHLQ. The sample size was determined to be 980 people, with 490 in the rural areas and 490 in the urban areas. The researchers visited potential participants at their doorstep, asking them to complete the questionnaire. The willing participants were assisted in completing the IHLQ in case they were illiterate; the questions and answers were read by the researchers to the participants, and the responses were recorded.

**Results:**

About 50.4% (*n* = 494) of the Kurdish population had poor health literacy, while 34.0% (*n* = 333) had average health literacy, and 15.6% (*n* = 153) had good health literacy. Meanwhile, 60.2% of the participants obtained poor scores in the construct of health information access, and 74.1% (*n* = 726) obtained poor scores in the individual empowerment construct. In addition, the analysis of the adjusted model indicated that education level (lowest β = 7.42; *P* = 0.001) and in male participants (β = − 1.10; *P* = 0.001) were significantly associated with higher health literacy.

**Conclusion:**

According to the results, the investigated Kurdish population mostly had average or low health literacy. Therefore, proper strategies should be adopted to enhance the health literacy of this population and increase their access to health information. Furthermore, effective training should be provided to these individuals (especially vulnerable social groups) to improve their individual capabilities to compensate for poor health literacy.

## Background

In the available literature, health literacy has been successfully used for the empowerment of various communities to promote self-care, confidence, and fair access to high-quality healthcare services [1–3]. Low health literacy is often prevalent in developing countries, such as Middle Eastern countries [4]. Health literacy has been investigated in different regions of Iran. In a study conducted on 1086 people in five provinces of Iran in 2007, 56.6% of the population had inadequate health literacy, and 15.3% had borderline health literacy [5]. Similarly, Ghanbari (2011) investigated the health literacy of the pregnant women referring to the primary healthcare centers, reporting that 30% of the pregnant women had inadequate health literacy, and 24% had borderline health literacy [6]. Another study conducted in 2012 indicated that inadequate health literacy was prevalent in the adult population, with a varied range in different regions of the country. According to the findings of Izadirad and Zareban, 41% of adults had inadequate health literacy in the central and more developed province of Yazd (Iran), while this rate was estimated at 68% in the adult population in the southeastern and less developed province of Baluchistan [7]. Moreover, a study conducted in 2015 indicated that only 18% of Iranians had adequate health literacy [8].

Kurdistan is one of the developing regions of Iran. Most of the inhabitants of Kurdistan province are Kurds, who represent one of the important ethnic minorities of Iran, as well as some other countries in the Middle East. In general, Kurdish communities live in various regions across the world. The health literacy of Kurdish populations seems to be understudied. Poor health literacy and health status seem to be more common among racial and ethnic minorities and in the developing parts of the world, which is the rationale behind the present study that aimed to measure the health literacy of the Kurdish population in Kurdistan province, Iran.

Poor health literacy negatively affects the economic, social, and health status of individuals [1]. Low health literacy is regarded as a public health concern, which follows a social gradient and may potentially exacerbate the current health inequalities [9]. Some of the main consequences of poor health literacy are increased health costs, mortality, and disease complications, which adversely affect social welfare [1, 3]. Furthermore, low health literacy is associated with poor self-assessed health status and difficulty in communication with healthcare professionals [9]. Low health literacy is also associated with the higher risk of hospitalization, readmission after discharge, and need for medical emergency services [10].

The definition of health literacy varies in in the extant literature, and each definition emphasizes a specific aspect of this concept. In general, health literacy is defined as the knowledge and qualification of an individual to manage the complicated requirements of health in the community [11]. According to the World Health Organization (WHO), health literacy is an important determinant of health, a lifestyle modification tool, and one of the main features of a healthy city. WHO urges all countries to promote health literacy in their strategic programs [2]. Therefore, the improvement of health literacy has become a major strategy of public health policies [3]. In fact, health literacy is a significant prerequisite for the prevention of non-communicable diseases, improving health and wellbeing, and reducing health inequality [2] and plays a pivotal role in the decision-making of individuals regarding health issues [12, 13].

Haghdoost et al. developed the Iranian health literacy questionnaire (IHLQ) in accordance with the health literacy definition given by Berkman and Davis [2, 8]. Since we used the IHLQ in our study [8], we also adhered to the health literacy definition by Berkman and Davis throughout the study. Correspondingly, health literacy is defined as the ability of individuals to gain, process, and understand the required health information and services and apply effective communication skills for appropriate healthcare decision-making [1].

The Association of Medical Specialists has classified the dimensions of health literacy into four categories, including conceptual and cultural literacy, oral literacy (speaking and listening), written literacy (reading and writing), and mathematical literacy [14]. In addition, they have introduced health knowledge as another dimension of health literacy [15]. It has been observed that low health literacy and poor health are correlated with deficient knowledge and understanding [10].

Exploring different perspectives on the dimensions of health literacy enriches our understanding of the concept. To date, various dimensions of health literacy have been studied, including reading and writing skills, understanding the concepts of basic health literacy [14, 16], ability to obtain health information [17], processing and realizing basic health services [18], ability to measure and identify health information [19], ability to transfer information to one’s health setting [20], ability to communicate with healthcare providers and decision-makers and performing the required tasks [17], understanding and exploring online health information [21], personal capabilities to use medical equipment and first aid [19], and cognitive and social capabilities and skills [22].

Currently, the issue of cultural differences and the need for the localization of health literacy measurement tools are emphasized worldwide [2, 3]. Measuring the health literacy of different populations within various contexts could provide valuable information to policymakers in order to make informed and evidence-based decisions [23]. Evidence-based policymaking regarding health literacy requires the assessment of health literacy levels in the general population [24]. Furthermore, improving the health literacy of the general population enhances the effectiveness of healthcare services [25].

This research strives to answer the question: what is the health literacy status of the Kurdish populations in Kurdistan province of Iran based on IHLQ nine constructs? Therefore, the present study aimed to assess the health literacy status of the Kurdish populations in Kurdistan province, Iran based on the nine constructs of the IHLQ, measure the health literacy of the population in terms of these constructs individually and collectively, and determine the significant effects of demographic variables on health literacy.

## Methods

### Study area

This cross-sectional study was conducted during April 2017–September 2018 in the urban and rural areas of Sanandaj (population: 463,681), Saqez (population: 363,681), and Qorveh (population: 156,350), which are the three main districts of Kurdistan province, located in the center, west, and east of the province. The sample population of the study was selected from the Kurdish-Iranian individuals aged more than 18 years who lived in Kurdistan province. The inclusion criteria were ability to complete the questionnaires or answer the questions orally, willingness to participate, and age of more than 18 years.

### Sampling

We used the $\mathrm{n}=\frac{{\mathrm{z}}^2.\mathrm{p}\left(1-\mathrm{p}\right)}{{\mathrm{d}}^2}$ formula at 95% confidence intervals (z = 1.98), margin of error of 0.02 (d), and prevalence (p) of 20%. In this formula, the prevalence was based on two relevant studies conducted in Iran regarding health literacy [3, 21]. Accordingly, the final sample size was calculated to be 980.

**Fig. 1:**
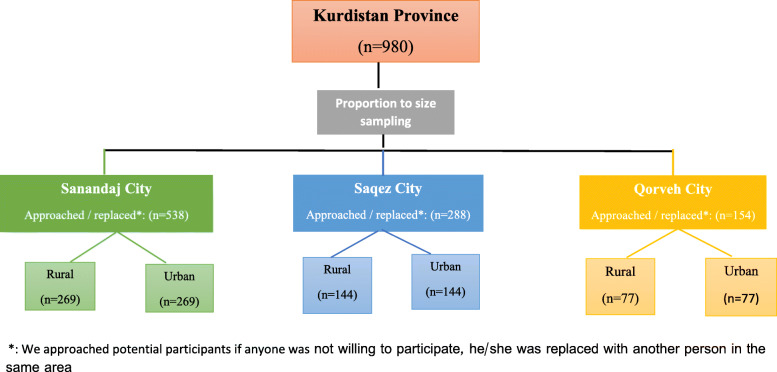
Sampling method diagram

The mentioned formula and the number of the participants ensured the fact that the research samples properly represented the statistical population of the study. The sample size was distributed equally from the rural and urban areas of the province since the population of the province is divided almost equally between the rural and urban areas. Multistage cluster sampling (systematic classification, clustering, and random methods) was applied to recruit 490 rural and 490 urban participants. The sample size in the selected cities was determined based on the population of each urban region and its rural areas proportionally (Fig. 1).

Based on the sample size of each area, a number of points were randomly selected on the map of the area, which served as the starting point of each cluster in data collection. The researchers attended the selected points, stood facing north, and started data collection from the first household on their right. Following that, data were collected from all houses continually until the samples of the cluster were completed. If a selected individual was not willing or available to participate, another person would be selected from the other households within the same cluster.

### Data collection

Data were collected on the sociodemographic characteristics and health literacy of the participants using a sociodemographic questionnaire and the IHLQ [8].

The sociodemographic questionnaire consisted of sex items (male/female), age (year), education level (illiterate, primary- junior high school, high school diploma, academic), place of residence (urban/rural), and employment status (student, homemaker, retired, unemployed, self-employed, part-time job, employed). The IHLQ was used to collect the health literacy data of the participants. According to Haghdoust et al. the IHLQ has nine constructs, including reading skills, comprehension skills, interpretation/judgment skills, communication/decision-making skills, individual empowerment, social empowerment, health knowledge, health information access and health information use. Haghdoust et al. have also confirmed the reliability and validity of the IHLQ [8].

The researchers visited potential participants at their doorstep, and verbal informed consent was obtained. In addition, the research objectives and procedures were explained, and the participants were assured of confidentiality and the right of withdrawal from the study at any given time. After obtaining informed consent, the questionnaires were completed by the participants who were able to do so without the help of the research team. However, the participants who were not confident or able to read and answer the questions were assisted by a member of the research team who would read out the questions and write down the answers.

### Data analysis

In order to calculate the total score of health literacy, the score of each construct was determined separately, and each construct was assigned a weight based on the number of its items. The weighted mean of health literacy was calculated by adding up the scores and dividing it by the number of the items per construct. In this process, a scale of 0–20 was considered, with the scores < 10, 10–14, and 14–20 indicating poor, average, and good health literacy, respectively. Notably, the same definition was adopted for the constructs of health literacy as well.

A manual was provided on finding and the recruitment of the participants and data collection in this study, which encompassed clear information on the selection of the regions and clusters and completion of the questionnaire. In addition, a questioner was assigned and trained for each area of data collection, and the descriptive analysis of the frequency, mean, standard deviation, and percentage of the collected data was also performed.

Data analysis was performed in SPSS version 22 using univariate and multivariable linear regression models, which were built to explore the determinants of health literacy. In addition, we introduce categorical variables to the multifactorial model by means of a set of indicators. For instance, education level had four categories, from which we selected one education level as the reference and developed three binary variables with the values of zero and one. To estimate the health Literacy scores of the Kurdish population, the clustering effect and sampling weights were computed and applied to the descriptive and analytical statistics using the random effects models. At this stage, age was considered as the covariate, and the other sociodemographic variables were the factor entered into the crude regression model; notably, the variables with the *P*-value of less than 20% were eliminated from the model. Finally, the adjusted regression model was employed with the remaining significant variables, and bias was controlled using the adjusted linear regression model, so that while observing the effect of one variable, the other variables of the model could remain constant.

## Results

### Characteristics of the participants

In total, 980 participants were enrolled in the study, with the mean age of 36.71 ± 11.88 years. Both sexes participated in the current research, including 50.9% males (*n* = 499). In terms of education level, 15.1% of the participants (*n* = 148) were illiterate, 43.3% had primary/junior high school education, 25.1% (*n* = 246) had a high school education or a high school diploma, and 16.5% (*n* = 162) had academic education.

With regard to occupation status, 42.1% of the participants (*n* = 413) were homemakers, 7.3% (*n* = 71) were unemployed, 3.9% (*n* = 38) were retired, 19.7% (*n* = 193) were self-employed, and 15.9% (*n* = 156) had a part-time job. Poor health literacy was observed in 22.6% of the students (*n* = 12), 62.5% of the homemakers (*n* = 258), 31.6% of the retired people (n = 12), 33.8% of the unemployed people (*n* = 24), 57.0% of the self-employed people (*n* = 110), 8.9% of the employed people (*n* = 5), and 43.5% of those with a part-time job (*n* = 68) (Table 1).

**Table 1:** Demographic characteristics of participants based on health literacy in the Kurdistan province in Iran, 2018

Demographic Characteristics	Total Participants n (%)	Poor Health literacy n (%)	Average literacy n (%)	Good Health literacy n (%)
Total Population	980 (100.0)	494 (50.4)	333 (34.0)	153 (15.6)
Mean (SD) age (years)	36.71 ± 11.88	39.56 ± 12.54	33.61 ± 10.71	33.00 ± 9.77
**Sex**				
Male	499 (50.9)	282 (56.5)	145 (29.1)	72 (14.4)
Female	481 (49.1)	216 (44.9)	182 (37.8)	83 (17.3)
**Education**				
Illiterate	148 (15.1)	147 (99.3)	1 (0.7)	0 (0.0)
Primary-junior high school	424 (43.3)	264 (62.3)	121 (28.5)	39 (9.2)
High school-diploma	246 (25.1)	68 (27.6)	120 (48.8)	58 (23.6)
Academic	162 (16.5)	25 (15.4)	86 (53.1)	51 (31.5)
**Place of Residence**				
Urban areas	490 (50.0)	239 (48.8)	184 (37.5)	67 (13.7)
Rural areas	490 (50.0)	257 (52.4)	147 (30.0)	86 (17.6)
**Employment**				
Student	53 (5.4)	12 (22.6)	28 (52.8)	13 (24.6)
Homemaker	413 (42.1)	258 (62.5)	112 (27.1)	43 (10.4)
Retired	38 (3.9)	12 (31.6)	21 (55.3)	5 (13.1)
Unemployed	71 (7.3)	24 (33.8)	32 (45.1)	15 (21.1)
Self-employed	193 (19.7)	110 (57.0)	59 (30.6)	24 (12.4)
Part-time job	156 (15.9)	68 (43.5)	63 (40.4)	25 (16.1)
Employed	56 (5.7)	5 (8.9)	20 (35.7)	31 (55.4)

Table 2 shows the frequency of the education level of the participants based on sex and place of residence. Accordingly, the illiteracy level of the women and residents of rural areas was higher compared to the men and residents of urban areas (Table 2).

**Table 2:** Frequency of participants’ education based on sex and residence in the Kurdistan province in Iran, 2018

Education level	Place of Residence		Sex		
	Urban areas n (%)	Rural areas n (%)	Male n (%)	Female n (%)	Total n (%)
Illiterate	47(9.6)	101(20.6)	42(9.0)	106(22.0)	148(15.1)
Primary-junior high school	149(30.4)	275(56.1)	216(43.4)	208(43.2)	424(43.3)
High school-diploma	150(30.6)	96(19.6)	138(26.7)	108(22.5)	246(25.1)
Academic	144(29.4)	18(3.7)	103(20.8)	59(12.3)	162(16.5)

### IHLQ results

In this study, health literacy was classified into three categories of poor health literacy (scores < 10), average health literacy (scores 10–14), and good health literacy (scores > 14). According to the findings, 99.3% of the illiterate people (*n* = 147), 62.3% of those with primary and junior high school education (*n* = 264), 27.6% of those with high school education and diploma (*n* = 68), and 15.4% of the people with academic education (*n* = 25) had poor health literacy. Among the participants with academic education, only 31.5% (*n* = 51) had good health literacy.

In the present study, the mean score of health literacy was 9.78 ± 0.13 (score range: 0–20). Approximately 50.4% of the Kurdish population (*n* = 494) had poor health literacy, 34.0% had average health literacy (*n* = 333), and only 15.6% had good health literacy (*n* = 153) (Table 1).

### Health literacy constructs

In the present study, poor scores were achieved by 74.1% (*n* = 726) and 60.2% of the participants (*n* = 590) in the constructs of individual empowerment and health information access, respectively. On the other hand, 42.1% (*n* = 413) of them achieved a good score in the construct of communication/decision-making skills. Table 3 shows the nine health literacy constructs in terms of the health literacy level of our participants.

**Table 3:** Health literacy levels based on the nine constructs among Iranian adult Kurdish population, 2018

Health literacy constructs	Poor health literacy n (%)	Average health literacy n (%)	Good health literacy n (%)
health information access	590 (60.2)	176 (18.0)	214 (21.8)
health information use	360 (36.8)	458 (46.7)	162 (16.5)
Reading skills	550 (56.1)	236 (24.1)	194 (19.8)
Comprehension skills	449 (45.8)	273 (27.9)	258 (26.3)
Interpretation / Judgment skills	385 (39.3)	291(29.7)	304 (31.0)
Communication /Decision-making skills	230 (23.5)	337 (34.4)	413 (42.1)
Health knowledge	391 (39.9)	206 (21.0)	383 (39.1)
individual empowerment	726 (74.1)	167 (17.0)	87 (8.9)
Social empowerment	436 (44.5)	247 (25.2)	297 (30.3)

According to the information in Table 4, the male participants achieved poor scores in some constructs of health literacy and average scores in the other constructs. The rural participants obtained higher health literacy scores in the constructs of communication/decision-making skills and health knowledge compared to the urban participants, while they achieved poor or average scores in the other constructs. Furthermore, the university and high school students achieved good health literacy scores in the construct of interpretation/judgment skills, and those with higher education levels obtained good health literacy scores in the other constructs, such as communication/decision-making skills.

**Table 4:** Demographic characteristics of participants based on health literacy constructs in the Kurdistan province in Iran, 2018

Demographic characteristics	health information access (Mean ± SD)	health information use (Mean ± SD)	Reading skills (Mean ± SD)	Comprehension skills (Mean ± SD)	Interpretation /judgment skills (Mean ± SD)	Communication /Decision-making skills (Mean ± SD)	Health knowledge (Mean ± SD)	Individual empowerment (Mean ± SD)	Social empowerment (Mean ± SD)
**Sex**									
Male	9.2 ± 4.8	9.6 ± 4.6	6.9 ± 6.6	8.7 ± 6.7	9.3 ± 7.0	12.6 ± 4.5	10.9 ± 5.8	4.5 ± 5.4	9.2 ± 5.8
Female	9.8 ± 5.3	9.8 ± 5.3	8.4 ± 6.5	10.3 ± 5.8	10.9 ± 5.9	13.0 ± 4.7	12.1 ± 5.9	5.9 ± 5.6	10.3 ± 6.0
**Education**									
Illiterate	7.3 ± 3.2	7.7 ± 3.8	0.0 ± 0.0	0.0 ± 0.0	0.0 ± 0.0	9.0 ± 4.8	7.2 ± 5.8	1.5 ± 3.4	5.6 ± 5.6
Primary-junior high school	8.5 ± 4.5	8.9 ± 4.5	7.1 ± 6.2	9.3 ± 5.2	9.8 ± 5.3	12.5 ± 4.5	11.2 ± 5.6	3.6 ± 4.5	9.7 ± 5.9
Junior-high school-diploma	10.8 ± 5.4	10.7 ± 5.2	10.6 ± 5.4	12.8 ± 4.7	13.4 ± 4.5	14.3 ± 3.9	13.3 ± 3.9	7.4 ± 5.6	11.0 ± 5.3
University	12.4 ± 5.5	12.1 ± 5.1	11.8 ± 5.6	13.7 ± 4.2	15.1 ± 4.4	14.8 ± 3.2	13.4 ± 5.1	10.0 ± 5.4	11.0 ± 5.3
**Residence**									
Urban areas	9.9 ± 5.4	10.3 ± 4.8	7.8 ± 5.9	9.6 ± 5.7	10.8 ± 6.0	12.6 ± 4.2	10.6 ± 5.7	6.0 ± 5.7	9.1 ± 5.3
Rural areas	9.3 ± 4.8	9.2 ± 4.9	7.6 ± 7.2	9.1 ± 6.9	9.4 ± 6.9	13.1 ± 4.9	12.6 ± 5.8	4.4 ± 5.1	10.5 ± 6.4
**Employment**									
Student	12.6 ± 5.0	11.6 ± 5.1	10.8 ± 5.5	13.3 ± 4.4	14.4 ± 4.5	13.5 ± 3.3	11.9 ± 5.5	8.6 ± 5.5	12.1 ± 4.4
Homemaker	8.6 ± 4.4	9.1 ± 4.3	6.1 ± 6.6	8.0 ± 6.7	8.5 ± 7.0	12.4 ± 4.5	10.7 ± 5.8	3.7 ± 4.8	8.7 ± 5.7
Retired	11.3 ± 4.9	11.7 ± 4.6	8.1 ± 5.2	11.1 ± 4.8	11.1 ± 5.1	15.1 ± 3.5	12.2 ± 5.9	7.3 ± 6.0	11.4 ± 5.5
Unemployed	10.5 ± 5.1	10.3 ± 5.4	9.8 ± 6.6	11.9 ± 5.4	12.8 ± 4.6	13.7 ± 4.0	13.7 ± 4.7	6.3 ± 5.2	11.7 ± 6.0
Self-employed	8.9 ± 5.1	9.2 ± 5.0	7.2 ± 6.4	8.9 ± 5.8	9.7 ± 6.1	12.0 ± 5.0	11.3 ± 6.0	4.9 ± 5.2	9.7 ± 6.1
Part-time job	9.2 ± 5.2	9.4 ± 5.0	8.8 ± 6.3	10.3 ± 5.5	11.0 ± 5.6	13.4 ± 4.4	12.7 ± 5.5	5.9 ± 5.4	10.6 ± 5.5
Employed	14.3 ± 5.9	13.5 ± 5.2	13.5 ± 5.7	14.8 ± 4.4	15.9 ± 4.2	16.0 ± 3.2	15.1 ± 4.6	12.2 ± 5.6	12.4 ± 5.4

Score < 10: Poor literacy.

Score 10–15: Average literacy.

Score > 14: Good literacy.

The findings of the current research indicated that the homemakers obtained poor scores in all the constructs of health literacy, with the exception of the health knowledge construct. In addition, the retired participants obtained good scores of health literacy in the construct of communication/decision-making skills, and the health literacy scores of the employees were also good in the constructs of health information access, comprehension skills, and communication/decision-making skills (Table 4).

In the present study, univariate and multivariate linear regression models were applied to determine the associations between the demographic characteristics of the participants with their total health literacy score. As anticipated, some of the findings differed in the crude and adjusted models (Table 5). In the crude model, the health literacy score of location and employment was not significant (*P* < 0.20), and these variables were not included in the adjusted model. Moreover, the crude score of health literacy decreased with increment in age, whereas it increased with age in the adjusted model although the finding was not considered significant (*P* = 0.098). In the crude model, the mean health literacy score of the men was lower than the women, while this was reversed in the adjusted linear regression model in which the mean health literacy score of the men was higher than the women (*P* = 0.001). Furthermore, a significant association was observed between the literacy level and health literacy level in the crude and adjusted models, and the association increased with the increasing literacy level (Table 5).

**Table 5:** Association between health literacy and independent variables in the crude and adjusted models among Iranian adult Kurdish population, 2018

Variable	Health literacy score	Simple linear regression (crude)		Adjusted	
		β	P-value	β	P-value
**Age**	9.78	0.11	0.001	0.016	0.098
**Sex**					
Male	9.22	–	–	–	–
Female	10.30	1.08	0.000	−1.10	0.001
**Education**					
Illiterate	4.13	–	–	–	–
Primary-junior high school	9.29	7.16	0.000	7.42	0.000
High school-diploma	11.74	8.79	0.000	8.87	0.000
Academic	12.92	8.79	0.000	8.93	0.000
**Employment**					
Unemployed	11.36	–	–	–	–
Student	12.21	0.84	0.193	–	–
Homemaker	8.62	−2.74	0.000	–	–
Retired	11.22	−0.139	0.847	–	–
Self-employed	9.37	−1.99	0.000	–	–
Part-time job	10.39	−0.974	0.061	–	–
Employed	14.40	3.04	0.000	–	–
**Place of Residence**					
Rural areas	09.67	–	–	–	–
Urban areas	09.88	0.20	0.425	–	–

## Discussion

Knowledge of health and the healthcare services is essential for the public. Health literacy plays a key role in self-care and health-related decision-making. According to the literature, various economic, cultural, and social factors affect the health literacy of different populations. Our findings could cultivate a deeper understanding of the influential factors in health literacy and health-related decision-making by our participants, as well as the population they represent in Kurdistan province.

The present study aimed to assess the constructs of health literacy in the Iranian adult Kurdish population, and the obtained results indicated that a vast majority of the Kurdish population had poor health literacy, which has implications for healthcare providers, healthcare policymakers, and the nongovernmental and governmental organizations concerned with the empowerment of communities. In the current research, 50.4% of the participants (*n* = 494) obtained poor health literacy scores. In the study by Haghdoust et al., 45.7% of the participants had inadequate health literacy [8]. Other studies conducted in Iran have also reported the low level of health literacy in the general population [26–28].

According to the results of the present study, 34.0% of the participants (*n* = 333) had average health literacy, and only 15.6% (*n* = 153) had good health literacy. This is consistent with the results obtained by Haghdoost et al., in which 36.3% of the participants had moderate health literacy, and 18% had adequate health literacy [8]. Furthermore, our findings in this regard are in line with the study by Tehrani Banihashemi et al. (2007), which was performed in five provinces of Iran [5]. Several other studies conducted in other countries have also confirmed the high prevalence of poor health literacy, estimating the value to be 26–68% [12]. In a study conducted in eight European countries (Austria, Bulgaria, Germany, Greece, Ireland, the Netherlands, Poland, and Spain), a public health challenge indicated that more than 10% of the population had poor health literacy, while approximately 29–62% had limited health literacy [29]. Although poor health literacy seems to be more prevalent in developing countries, Paasche-Orlow reported the inadequate health literacy of the general population in the developed countries of North America [12]. However, Johri et al. concluded that it is possible to modify health literacy based on novel interventions within short periods [30].

Tehrani Banihashemi and Paasche-Orlow have reported no correlation between low health literacy and sex [5, 12]; while our findings indicated that health literacy was significantly lower in the female in the adjusted model compared to the male. This is consistent with some of the studies conducted in Iran [1, 29]. Low health literacy among women in the present study could be due to lower education level and employment in the women compared to men.

In another study, Kohan et al. observed no direct correlation between education level and health literacy [1], while the results of the present study demonstrated that health literacy increased with the higher education level of the participants. This is in line with some of the studies conducted in Iran and other studies [6, 25, 29, 31–34]. Moreover, the present study indicated that the people with higher education levels obtained a higher score in the health literacy construct of communication/decision-making skills.

In the current research, poor health literacy among the homemakers and villagers could be attributed to some social factors. Poor health literacy is associated with poor socioeconomic status and complex health inequalities [24]. In fact, only 2.7% of the villagers had academic education, and 76.2% of the homemakers were illiterate. Furthermore, the homemakers had low health literacy scores in all the constructs, with the exception of health knowledge. In a similar study, social factors (e.g., low educational attainment) were associated with the inadequate health literacy of British adults [35], as well as in other European communities [29]. In addition, the study conducted by Tavousi showed that homemakers had poor health literacy [26].

According to the results of the present study, the retired participants had good health literacy scores in the construct of communication/decision-making skills, while the employees obtained good scores in terms of health information access and communication/decision-making skills. On the same note, Ghanbari et al. reported that health literacy promoted with transition from homemaking to employment [6]. Using social media and other mediums (e.g., cultural events) may help older adults to preserve health literacy skills while ageing [36].

In the present study, the analysis of the nine constructs of health literacy indicated that 60.2% (*n* = 590) and 74.1% of the participants (*n* = 726) obtained poor health literacy scores in the constructs of health information access and individual empowerment, respectively. Therefore, new strategies should be adopted to provide access to health information and proper training to improve the abilities of the general population in this regard. The improvement of health literacy in the communities with poor socioeconomic status could yield remarkable public health benefits [35], and this strategy must be primarily focused on vulnerable populations, such as villagers, women, and homemakers. Our findings have some implications for the Iranian Kurdish population, and the generalization of the findings to other ethnicities should be considered with percussions.

The main limitations of the present study were the study setting and the cultural and socioeconomic differences in various regions of Kurdistan province, which were partially reversed through the randomized selection of the data collection areas and determining an appropriate sample size.

## Conclusion

This study aimed to evaluate the health literacy of the Kurdish population in Iran. According to the results, a high percentage of the participants had poor or average health literacy. Therefore, it seems essential to adopt strategies to enhance health literacy in Kurdistan province. We also attempted to assess the associations between the constructs of health literacy and demographic factors, and the findings indicated that the health literacy scores were lower among women, less educated people, homemakers, and villagers. Therefore, implementing interventional strategies to improve the health literacy of these vulnerable social groups also seems necessary.

According to our findings, the participants obtained poor scores in the construct of health information access, which highlights the need for the mass media to disseminate health information efficiently and promptly. Healthcare providers, health insurance companies, and social policymakers could also benefit from improving the health literacy of communities, which becomes more pronounced in the case of underprivileged and vulnerable social groups.

## Data Availability

The datasets used and/or analyzed in the study are available from the corresponding author on reasonable request.

## Abbreviations

WHO: World Health Organization
IHLQ: Iranian Health Literacy Questionnaire
